# Heterologous expression, purification and biochemical characterization of a glutamate racemase (MurI) from *Streptococcus mutans* UA159

**DOI:** 10.7717/peerj.8300

**Published:** 2019-12-20

**Authors:** Xiangzhu Wang, Chanchan Chen, Ting Shen, Jiangying Zhang

**Affiliations:** 1 Department of Operative Dentistry and Endodotics, Xiangya School of Stomatology, Xiangya Stomatological Hospital, Central South University, Changsha, Hunan, China; 2 Department of Stomatology, Shenzhen Children’s Hospital, Shenzhen, Guangdong, China

**Keywords:** *Streptococcus mutans*, MurI, Prokaryotic expression, Affinity chromatography, Glutamate racemase, Enzymatic reaction, Peptidoglycan biosynthesis

## Abstract

**Background:**

Glutamate racemase (MurI) is a cofactor-independent enzyme that is essential to the bacterial peptidoglycan biosynthesis pathway and has therefore been considered an attractive target for the development of antimicrobial drugs. While in our previous study the essentiality of the *murI* gene was shown in *Streptococcus mutans*, the primary aetiologic agent of human dental caries, studies on *S. mutans* MurI have not yet provided definitive results. This study aimed to produce and characterize the biochemical properties of the MurI from the *S. mutans* UA159 genome.

**Methods:**

Structure characterization prediction and multiple sequence alignment were performed by bioinformatic analysis. Recombinant His_6_-tagged *S. mutans* MurI was overexpressed in the expression vector pColdII and further purified using a Ni^2+^ affinity chromatography method. Protein solubility, purity and aggregation state were analyzed by SDS–PAGE, Western blotting, native PAGE and SEC-HPLC. Kinetic parameters were assessed by a circular dichroism (CD) assay. Kinetic constants were calculated based on the curve fit for the Michaelis–Menten equation. The effects of temperature and pH on enzymatic activity were determined by a series of coupled enzyme reaction mixtures.

**Results:**

The glutamate racemase gene from *S. mutans* UA159 was amplified by PCR, cloned and expressed in *Escherichia coli* BL21 (DE3). The 264-amino-acid protein, as a mixture of dimeric and monomeric enzymes, was purified to electrophoretic homogeneity. In the CD assay, *S. mutans* MurI displayed unique kinetic parameters (*K*_m, d-Glu→l-Glu_ = 0.3631 ± 0.3205 mM, *V*_max, d-Glu→l-Glu_ = 0.1963 ± 0.0361 mM min^−1^, *k*_cat, d-Glu→l-Glu_ = 0.0306 ± 0.0065 s^−1^, *k*_cat_/*K*_m,__d-Glu→l-Glu_ = 0.0844 ± 0.0128 s^−1^ mM^−1^, with d-glutamate as substrate; *K*_m, l-Glu→d-Glu_ = 0.8077 ± 0.5081 mM, *V*_max, l-Glu→d-Glu_ = 0.2421 ± 0.0418 mM min^−1^, *k*_cat_*_,_*_l_*_-_*_Glu→d-Glu_ = 0.0378 ± 0.0056 s^−1^, *k*_cat_/*K*_m,__l-Glu→d-Glu_ = 0.0468 ± 0.0176 s^−1^ mM^−1^, with l-glutamate as substrate). *S. mutans* MurI possessed an assay temperature optimum of 37.5 °C and its optimum pH was 8.0.

**Conclusion:**

The findings of this study provide insight into the structure and biochemical traits of the glutamate racemase in *S. mutans* and supply a conceivable guideline for employing glutamate racemase in anti-caries drug design.

## Introduction

Dental caries is one of the most common chronic diseases noted throughout the world and is a disease caused by dysbiosis in the oral cavity (Lamont, Koo & Hajishengallis, 2018). If left untreated, dental caries can lead to tooth defects, pain, infection, and, in severe cases, tooth loss. Dental caries has always been a major global health concern for professionals because of their adverse impact on overall health in how they affect nutrient utilization (Pitts et al., 2017).

*Streptococcus mutans*, a gram-positive diplococcus that is found mainly in oral biofilms, has evolved into a normal resident of the oral flora but has long been implicated as the causative pathogen for human dental caries (Zhou et al., 2018). It is paramount to identify and validate more potential targets that influence the virulent cariogenic traits of *S. mutans* for anti-caries therapeutic purposes due to the dominant role that *S. mutans* plays in the initiation and progression of caries lesions (Loesche, 1986).

The cell wall is the outermost barrier between the cell and the environment, protecting the cell from the environment and playing a crucial role in a number of biological processes vital for bacterial growth and survival (Bugg et al., 2011). Peptidoglycan is the main component of the cell wall. The biosynthesis of peptidoglycan is a complicated process that is separated into three stages with distinctive cellular localizations. The pathway begins (stage I) with the synthesis of nucleotide precursors in the cytoplasm. The second stage, the synthesis of lipid-linked intermediates, is performed on the internal face of the cytoplasmic membrane (CM), while stage III functions (polymerization reaction) occur outside the CM (Bugg et al., 2011). Most active drugs, such as β-lactams and moenomycin, repress the phase III pathway, which involves the extracellular crosslinking and development of the cell envelope (Silver, 2013). However, phase I appears to be a rich source of attractive enzyme targets for new antibacterials. Therefore, targeting enzymes involved in phase I, such as alanine racemase, glutamate racemase, and GlmS, may represent a promising new strategy in the discovery of antibacterials (Kotnik, Anderluh & Prezelj, 2007).

Glutamate racemase (E.C. 5.1.1.3) is a cofactor-independent enzyme in microorganisms that is capable of catalyzing the interconversion of l-glutamate to d-glutamate, an indispensable element of the pentapeptide that crosslinks the glycan strands of peptidoglycan (Hernández & Cava, 2016). Two types of glutamate racemase have been recognized within the cytoplasm of microbes: the *murI*-encoded racemase (MurI) and the D-amino acid aminotransferase (*daaT*)-encoded racemase (DaaT) (Fisher, 2008). In *Mycobacterium smegmatis*, whose genome carries solely *murI*, genetic deletion studies have indicated that the lack of MurI eliminates growth in the absence of external d-glutamate supplementation (Li et al., 2014). Other bacteria, such as *Bacillus sphaericus*, have another enzyme (DaaT) that can deliver adequate d-glutamate in the absence of MurI (Kimura, 2004). MurI is ubiquitous in most bacteria but lacks a human homolog (Silver, 2013), which renders the enzyme an attractive drug target for the inhibition of cell wall biosynthesis in species including *Helicobacter pylori* (De Jonge et al., 2015), *Mycobacterium tuberculosis* (Prosser et al., 2016), *Staphylococcus aureus* (Whalen, Chau & Spies, 2013), and *Escherichia coli* (Doublet et al., 1993).

In our previous study, a *murI* mutant strain of *S. mutans* UA159, whose genome contained only *murI*, was constructed. Our data demonstrated that the *murI* gene was essential for virulence and environmental stress tolerance in this streptococcal species. The depletion of *murI* led to overall malfunction as well as differences in cell morphology in Δ*murI* cells (Zhang et al., 2016). The complete genome sequence of *S. mutans* paves the way for determining its protein sequences. These proteins can be further explored for their use as potential drug targets using various in silico approaches, thereby allowing in-depth identification of potent candidates using high-throughput screening methods. In fact, several different drugs have been reported as MurI inhibitors, such as ethambutol (Pawar et al., 2019), L-serine O-sulfate (Vidya et al., 2008), and pyrazolopyrimidinediones (De Jonge et al., 2009). Recently, glutamate racemases from pathogenic and nonpathogenic microorganisms have been recombinantly expressed in an active form in *E. coli*, purified by precipitation, ion exchange chromatography, and His-tag affinity chromatography and subsequently characterized (Böhmer et al., 2013). In the present study, we focused on the glutamate racemase from *S. mutans* UA159 serotype *c* (a proven virulent strain causing caries). The glutamate racemase from *S. mutans* UA159 was investigated for recombinant production in *E. coli* BL21 (DE3). Additionally, the purified enzyme was biochemically elucidated.

## Materials and Methods

### Bioinformatic analysis of a putative glutamate racemase in *S. mutans* UA159

The amino acid sequence of MurI was obtained from the National Center for Biotechnology Information (NCBI) (AAN59353.1). A 3-dimensional (3D) model of MurI was built (*Streptococcus pyogenes* serotype M1 was used as a template, Swiss-Prot: SMTL ID: 2ohg.1) by SWISS-MODEL using a standard procedure and was analyzed. After obtaining the 3D model, the possible active centers of glutamate racemase were analyzed and generated automatically by SWISS-MODEL (https://swissmodel.expasy.org/repository/uniprot/Q8DSQ5?csm=C9E0C894C19E1760). Multiple amino acid sequences were aligned using ESPript 3.0 (http://espript.ibcp.fr/ESPript/cgi-bin/ESPript.cgi) and the ClustalX program. A phylogenetic tree was inferred from the alignments using the neighbor-joining method and was drawn using the TreeView program (http://www.treeview.net/).

### Plasmids, bacterial strains, and growth conditions

Unless otherwise stated, laboratory chemicals, reagents, and disposable lab ware were purchased from Sigma–Aldrich (St. Louis, MO, USA), Thermo Fisher Scientific (Waltham, MA, USA), or Oxoid (Basingstoke, Hampshire, UK). *S. mutans* UA159 (ATCC700610) (Guangdong Culture Collection Centre of Microbiology, Guangzhou, China) was used as the source of chromosomal DNA for the polymerase chain reaction (PCR). *S. mutans* UA159 cells were cultured in BHI broth (Difco, Sparks, MD, USA) at 37 °C under anaerobic conditions (5% CO_2_, 10% H_2_, and 85% N_2_). The *E. coli* strains DH5α and BL21 (DE3) were used as hosts for gene manipulation and expression procedures. The *E. coli* strains were grown in LB broth (Difco, Sparks, MD, USA) and plated onto the LB medium containing 1.5% (w/v) agar at 37 °C. Ampicillin was added when required to a final concentration of 100 μg mL^−1^.

### Construction of a recombinant plasmid carrying MurI

The *S. mutans murI* gene was amplified from genomic DNA using the PCR system with the appropriate primers IGLUT-F and IGLUT-R (Table 1). The amplicon was cloned into the pColdII expression vector to create the construct pColdII-*murI* with a His tag using a NovoRec^®^ PCR one-step cloning kit (Novoprotein, Summit, NJ, USA) according to the manufacturer’s instructions. The resulting recombinant pColdII-*murI* with a His tag was sequenced for confirmation (using the primers pCold II-F and IGLUT-R) and then transferred into *E. coli* BL21 (DE3) cells for production of the His_6_-tagged (N-terminus) *S. mutans* MurI protein.

**Table 1:** Bacterial strains and plasmids used in this study. All primers were designed with primer-BLAST and obtained from Invitrogen Biotechnology.

Bacterial strains	Major properties	Source or reference
*S. mutans* UA159	Wild type, serotype *c*, virulent strain causing caries	Ajdić et al. (2002)
*E. coli* DH5α	*endA1, hsdR17, supE44, recA1* (*lacZYA-argF*)	Takara
*E. coli* BL21 (DE3)	*F-, ompT, hsdS* (*rBB-mB-*), *gal, dcm* (*DE3*)	Novagen
Plasmids		
pColdII	Expression vector, ampicillin resistance (Amp^r^)	Novagen
pCold-murI	795-bp DNA fragment of the glutamate racemase nucleotide sequence cloned into *Nde*I–*Hin*dIII sites of pColdII, Amp^r^	This work
**Primers**	**DNA sequences (5′-3′)**	**Purpose**
IGLUT-F^a^	GTGCATCATCATCATCATCATATGGATAATCGTCCGATTGGTTTTC	PCR
IGLUT-R^a^	AGACTGCAGGTCGACAAGCTTTCACAGGGTAACATGTTCAAC	PCR and sequencing
pCold II-F	ACGCCATATCGCCGAAAGG	Sequencing

**Notes:**

a Nucleotides underlined are restriction enzyme sites engineered for cloning.

### Expression and purification of *S. mutans* MurI

The pColdII-*murI* construct was introduced into *E. coli* BL21 (DE3) by heat shock transformation as described in the manufacturer’s protocol. The transformed cells were grown in 1 L of LB medium containing 50 µg mL^−1^ ampicillin at 37 °C until the OD_600_ reached 0.6–0.8. The expression of *S. mutans* MurI in *E. coli* BL21 was induced with 0.5 mM isopropyl-β-d-thiogalactoside (IPTG). After further incubation for 6 h at 15 °C, cell pellets were collected, and transmission electron microscopy (TEM) was used to detect inclusion body formation. The aggregated pellets were resuspended in a lysis buffer (50 mM Tris, 150 mM NaCl, 5% glycerol, pH 8.0) and sonicated. The supernatant fluid was kept for future purification after centrifugation (21,130×*g* for 10 min). All soluble recombinant proteins were purified on a Ni Sepharose 6 Fast Flow column (GE Healthcare, Piscataway, NJ, USA) according to the manufacturer’s instructions. The affinity column was pre-equilibrated with a binding buffer (50 mM Tris-HCl, 150 mM NaCl, 20 mM imidazole, pH 8.0). After the column was washed with binding buffer to remove the unbinding proteins, the bound *S. mutans* MurI was eluted by a linear gradient of imidazole from 50 mM to 500 mM in an elution buffer (50 mM Tris-HCl, 150 mM NaCl, pH 8.0). The eluted fractions were dialyzed overnight at 4 °C against 100 mM NaCl, and then ultrafiltrated using an Amicon^®^ Ultra (NMWL; 30 kDa, Millipore, Burlington, MA, USA) at 14,000×*g* for 30 min at 4 °C. The purity and specificity of *S. mutans* MurI was verified by sodium dodecylsulfate–polyacrylamide gel electrophoresis (SDS–PAGE) and Western blot analysis using a mouse anti-His tag monoclonal antibody (Abgent, San Diego, CA, USA). Protein stocks were stored in 10% glycerol at −80 °C until use at a concentration of 2 mg mL^−1^.

### Non-denaturing polyacrylamide gel analysis

The soluble recombinant *S. mutans* MurI was subjected to non-denaturing polyacrylamide gel electrophoresis (native PAGE) as described previously (Zhuang et al., 2016), with modifications. The proteins were loaded onto 6.5% Bis–Tris gels and resolved by electrophoresis at 4 °C. The gels were stained with BeyoBlue^™^ Coomassie Blue staining solution (Beyotime, Haimen, Jiangsu, China), and the protein bands were visualized. The dimer/monomer ratio of MurI proteins was assessed by means of comparing the densities of dimer and monomer bands using ImageJ software (National Institutes of Health, Bethesda, MD, USA). The reported ratios are the means of triplicate assays.

### Size-exclusion-high-performance liquid chromatography analysis

Size-exclusion-high-performance liquid chromatography (SEC-HPLC) of intact proteins under native conditions was carried out to determine the native molecular mass of glutamate racemase on an Agilent 1,260 Infinity II HPLC system equipped with an Agilent AdvanceBio SEC 300 Å column (4.6 mm × 300 mm, 2.7 μm). Isocratic elution of MurI samples was conducted using a mobile phase of 100 mM phosphate buffer containing 100 mM sodium sulfate (pH 6.7) at a flow rate of 0.3 mL/min. The column temperature was maintained at 25 °C, and the column eluent was monitored and detected at a wavelength of 280 nm. Data was acquired and processed using Agilent MassHunter Qualitative Analysis software. The relative percentage contents were calculated by the area normalization method.

### Determination of kinetic parameters using circular dichroism assay

The kinetic parameters of *S. mutans* MurI were analyzed for the substrates l-glutamate and d-glutamate. A circular dichroism (CD) assay was used, whereby the change in ellipticity was monitored using a Jasco J-815 CD spectrophotometer (JASCO International Co., Tokyo, Japan), according to the method described by Noda et al. (2005) with some modifications. Reactions were conducted in 10 mM potassium phosphate buffer (pH 7.8) at 37 °C with substrate concentrations of 0.3125–10 mM in a final volume of 200 μL in a 0.1 cm quartz cuvette. The reaction was started by addition of the purified MurI (dialyzsed against 10 mM potassium phosphate buffer (pH = 7.8) and at a final concentration of 6.25 μg/mL). The change in ellipticity at 202 nm for d-glutamate and 204 nm for l-glutamate were measured over a period of 5 min. Spectra were averaged from three scans and recorded between 190 and 230 nm by 0.05 data pitch. The resulting spectra were treated without smoothing. Velocities were determined using a molar ellipticity ([θ]) of 31.0 mdeg mM^−1^ cm^−1^ for l-glutamate and 29.0 mdeg mM^−1^ cm^−1^ for d-glutamate, respectively, in reasonable agreement with the previously reported values of 31.0 mdeg mM^−1^ cm^−1^ for glutamate (May et al., 2007). The values of the kinetic constant were determined by fitting initial velocity (*v_i_*) data to Eq. (1) using nonlinear regression and the software Graphpad Prism v 5.0 (GraphPad Software Inc., San Diego, CA, USA).

(1) $v_i = V_{\rm max}\left[ S \right]/\left( {K_{\rm m} + \left[ S \right]} \right)$

where *v*_i_ is the slope during the linear phase of cleavage and (*S*) is the substrate concentration. The catalytic rate constant (*k*_cat_) was calculated based on the ratio of *V*_max_ to the total concentration of the enzyme, using 58,528 Da as the molecular weight (MW) value for the dimer, and the catalytic efficiency was determined based on the *k*_cat_/*K*_m_ ratio. All reported values are the means of triplicate assays.

### Effect of temperature and pH on *S. mutans* MurI activity

In the set of experiments, an enzyme-coupled reaction was used to measure the enzymatic activity of MurI with d-glutamate as the substrate based on the method described (Gallo & Knowles, 1993; Liechti et al., 2018) with some modifications. Briefly, the MurI activity was measured in the d- to l-glutamate direction by monitoring the production of NADH at 340 nm as the l-glutamate produced was converted to α-ketoglutarate and ammonia by l-glutamate dehydrogenase (LGDH). Reactions (the first-step reaction) were performed in a final volume of 0.1 mL reaction buffer containing 0.1 M Tris-HCl (pH 8.0), 10 mM d-Glu, 0.5 mM EDTA, and 20 μg of purified MurI. Reaction mixtures were incubated at 37 °C for 20 min and then heat inactivated at 100 °C for 5 min prior to the l-Glu assay. The l-Glu assay (the second-step reaction) mixture with a total volume of 0.1 mL contained 0.1 M Tris-HCl (pH 8.0), 5 mM NAD^+^ (MCE, Monmouth Junction, NJ, USA), and 50 μl of the first reaction mixture. The OD_340_ was monitored after the addition of 20 units of LGDH from bovine liver (Sigma–Aldrich, St. Louis, MO, USA) after 10 min incubation at 25 °C in 96 well clear microplates using a Varioskan 3001 Scanning Multimode Microplate Reader (ThermoFisher Scientific, Waltham, MA, USA). The effect of temperature on the activity of recombinant MurI was investigated by measuring the enzymatic activity as described above in the first-step reaction, varying the temperatures from 12.5 to 100 °C. The effects of temperature on enzyme stability were also examined; *S. mutans* MurI was pre-incubated in 0.1 M Tris-HCl (pH 8.0) at 0, 20, 40, 60, and 80 °C for 60 min, and then the remaining activity was measured under the standard assay conditions. The effect of pH on the activity of recombinant MurI was examined with three different buffers in the pH range of 6.0–10.5. The buffers 0.1 M glycine-NaOH (pH 9.5–10.5), 0.1 M Tris-HCl (pH 7.5–9.0), and 0.1 M potassium phosphate (KH_2_PO_4_/K_2_HPO_4_, pH 6.0–7.0) were used in the experiment. The relative activity was calculated, using the sample with the highest activity as 100%.

## Results

### Sequence analysis of the putative glutamate racemase (MurI) gene from *S. mutans* UA159

**Figure 1:**
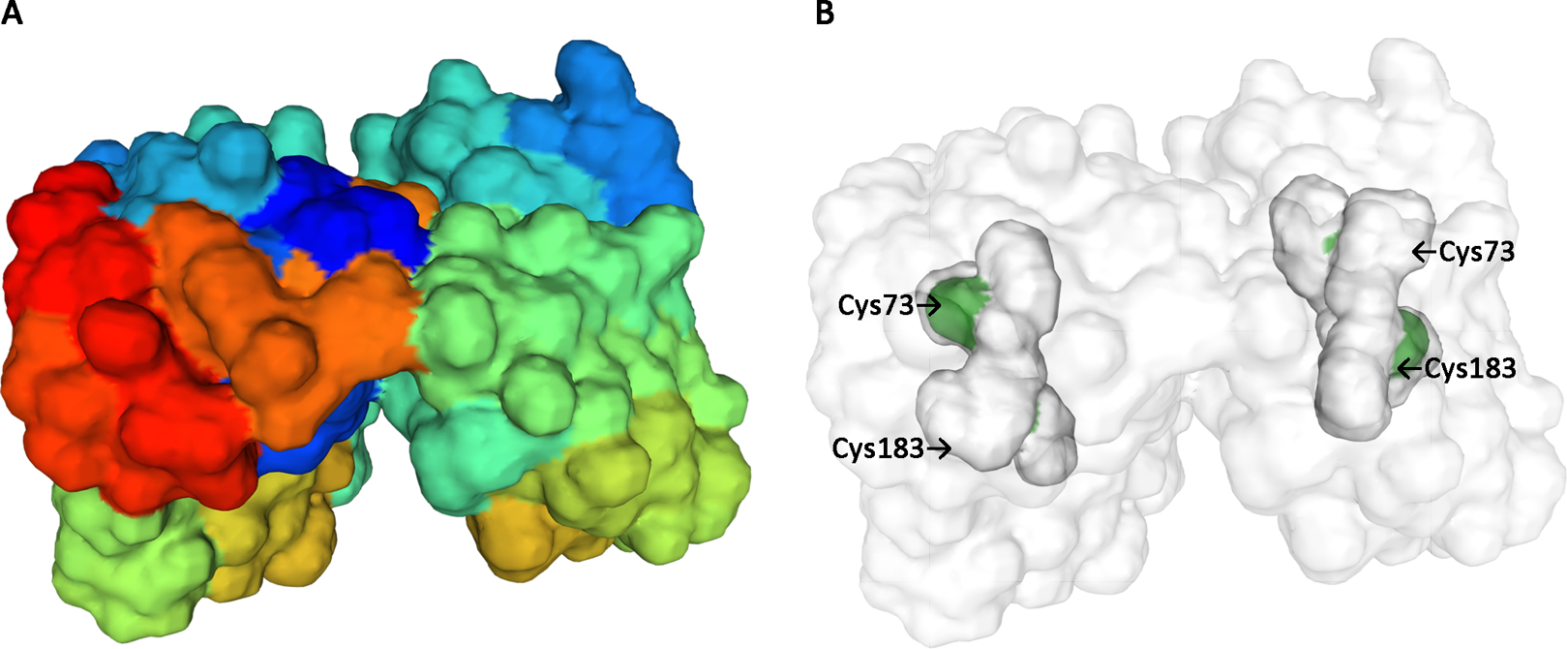
Glutamate racemase (MurI) in *Streptococcus mutans* UA159. (A) Construction of a 3D model of MurI using the *Streptococcus pyogenes* serotype M1 (SMTLID: 2ohg.1) enzyme as a template. The template and the target have 78.41% identical residues, with a QMEAN-value of −1.01. (B) Two important cysteine residues of the active center for the proton donor/acceptor in MurI, Cys73 and Cys183.

**Figure 2:**
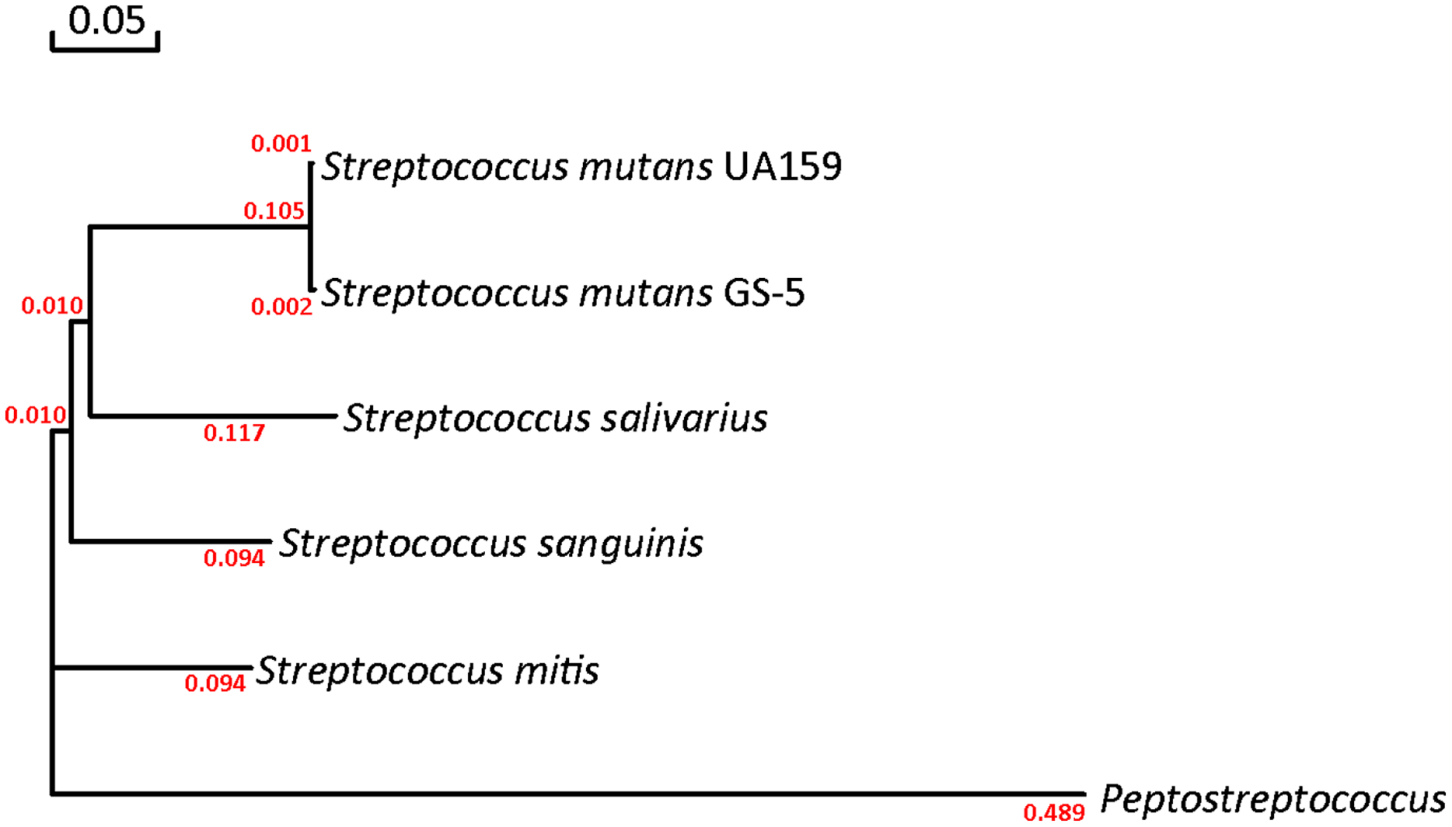
Phylogenetic tree based on the amino acid sequence of MurI. Sources and GenBank accession numbers are as follows: *S. mutans* UA159, AAN59353.1; *S. mutans* GS-5, AFM82044.1; *S. salivarius*, KEO46897.1; *S. sanguinis*, WP_002894602.1; *S. mitis*, KER08828.1; and Peptostreptococcus, WP_002842877.1. Scale bar: evolutionary distance of 0.05 amino acid residues per position in the sequence. Branch length scales are shown at the base of the tree.

The protein sequence of glutamate racemase from *S. mutans* UA159 was obtained from the NCBI database (Accession number AAN59353.1, https://www.ncbi.nlm.nih.gov/protein/24378087/) and was inferred through homology to be the putative glutamate racemase (MurI). Analysis of the DNA sequence revealed that the *murI* open reading frame was 795–bp long and encoded a 264-amino-acid protein with a calculated molecular mass of 29,264 Da and an isoelectric point of 8.85. The 3D structure topology of MurI resembled that of *S. pyogenes* serotype M1 (SMTL ID: 2ohg.1; Fig. 1A; Fig. S1), indicating that MurI is a homodimer and carries the two catalytic amino acid residues (Cys83 and Cys173) of the active center (Fig. 1B). Comparison and analysis of the protein sequence encoded by *murI* from *S. mutans* UA159 revealed a close identity with the protein sequence of MurI from *S. mutans* GS-5 (Accession number AFM82044.1; 99% identity). Moreover, *S. mutans* UA159 MurI displayed a different level of similarity with the MurIs in other known cariogenic oral streptococci (Fig. S2). To assess the phylogenetic relationship between UA159 MurI and other MurIs, we aligned their amino acid sequences and constructed a phylogenetic tree (Fig. 2). The analysis revealed that the enzyme from *S. mutans* UA159 clustered with that of other streptococcal species. Intriguingly, MurI from *Peptostreptococcus* was located in a cluster distinct from the one containing the MurIs from the five gram-positive bacteria, which indicated that it is evolutionarily different from these MurIs.

### Cloning, expression, and purification of glutamate racemase

**Figure 3:**
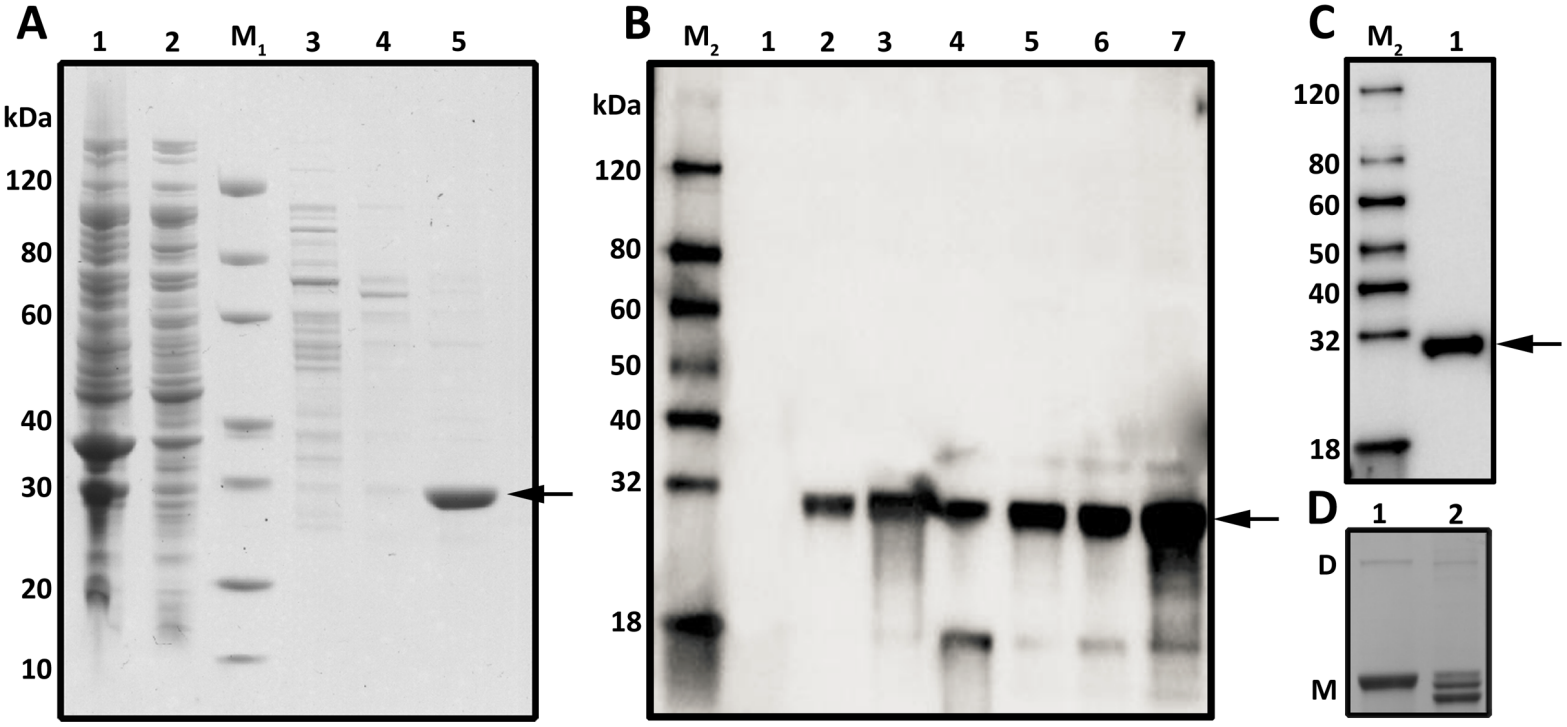
SDS–PAGE, Western blot and Native PAGE analysis of the expression and purification of MurI in *E. coli* BL21 (DE3). (A) SDS–PAGE analysis of the purification process of MurI in *E. coli* BL21 (DE3). Detailed legend: Lane M1: Protein marker. Lane 1: Supernatant after cell lysate centrifugation (loading). Lane 2: Flow through. Lane 3: Wash with 50 mM Tris-HCl, 150 mM NaCl, 20 mM imidazole, pH 8.0. Lane 4: Elute with 50 mM Tris-HCl, 150 mM NaCl, 50 mM imidazole, pH 8.0. Lane 5: Elute with 50 mM Tris-HCl, 150 mM NaCl, 500 mM imidazole, pH 8.0. (B) Western blot analysis of non-purified MurI probed with mouse anti-His monoclonal antibody. Detailed legend: Lane M2: Western marker. Lane 1: Cell lysate without induction. Lane 2: Cell lysate with induction for 16 h at 15 °C. Lane 3: Cell lysate with induction for 4 h at 37 °C. Lane 4: Supernatant of cell lysate with induction for 16 h at 15 °C. Lane 5: Debris of cell lysate with induction for 16 h at 15 °C. Lane 6: Supernatant of cell lysate with induction for 4 h at 37 °C. Lane 7: Debris of cell lysate with induction for 4 h at 37 °C. (C) Western blot analysis of purified MurI probed with mouse anti-His monoclonal antibody. Detailed legend: Lane 1: Purified MurI. The estimated MW of MurI was approximately 31 kDa, as indicated by the arrows. (D) Native PAGE analysis of purified MurI in native status. Detailed legend: Lanes 1: Protein stored in PBS, 10% glycerol, pH 7.4. Lanes 2: Protein stored in 50 mM Tris-HCl, 150 mM NaCl, 10% glycerol, pH 8.0. The band labeled with D indicates the band of dimeric protein; the band labeled with M indicates the band of monomeric protein.

pColdII-*murI* was transformed into *E. coli* BL21(DE3), and the expression of *S. mutans* MurI as a recombinant protein was induced by IPTG. Under TEM observation, there were no aggregates (inclusion bodies) formed in the cytoplasmic or periplasmic area during IPTG induction (Fig. S3).The recombinant His-tagged MurI was subsequently purified using Ni Sepharose and analyzed by SDS–PAGE and Western blot (Fig. 3; Fig. S4). A total of 1.14 mg of purified recombinant MurI was obtained from 1 L of *E. coli* culture. Figure 3A shows the SDS–PAGE profiles of the MurI preparations obtained at each purification step. Most protein contaminants were removed after one-step affinity chromatography eluted by a linear gradient of imidazole (Fig. 3A, lanes 3 and 4). The purified MurI migrated as a single band on an SDS–PAGE gel (Fig. 3A, lane 5). As depicted in Fig. 3B, Western blot analysis revealed that the target protein was expressed in the whole cell lysate, supernatant, and debris of the cell lysate, indicating that the *murI* gene was functionally expressed in *E. coli*. The protein solubility was estimated to be ~50%. Examination of the expressed products showed that the subunit MW of His-tagged MurI was approximately 31 kDa, which is slightly higher than the theoretical molecular mass calculated from the amino acid sequence in silico. Western blot analysis of MurI probed with a mouse anti-His tag monoclonal antibody revealed anti-His antibody-reactive bands (Fig. 3C). Then, native PAGE was used to evaluate the native status of purified MurI. As shown in Fig. 3D, recombinant MurI primarily existed in a combination of dimer and monomer (predominant) forms. The monomer/dimer ratio of MurI was approximately 2.03 ± 2.08.

### Molecular mass determination by SEC-HPLC

**Figure 4:**
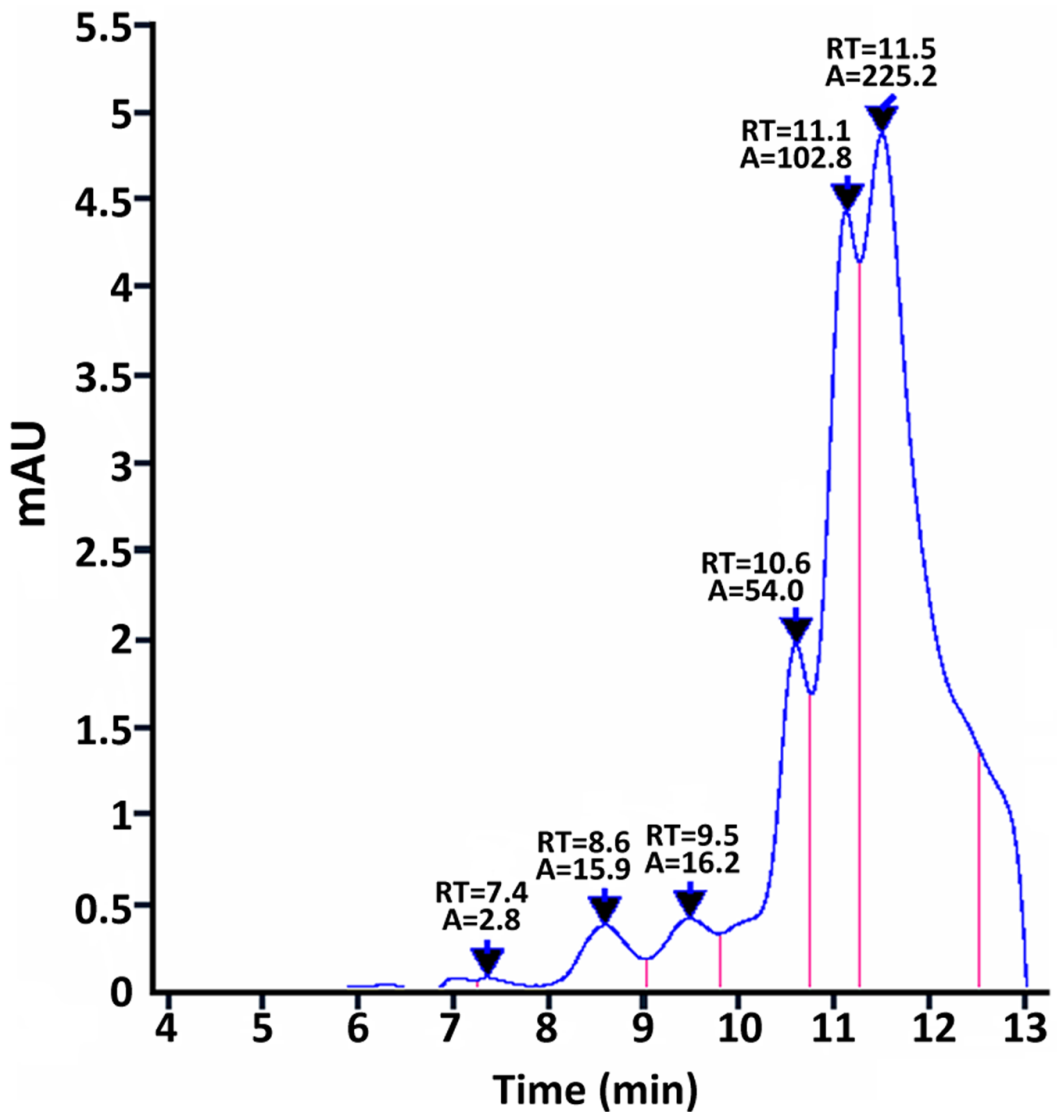
SEC-HPLC analysis of purified MurI under native conditions. The relative amount of the main form of monomeric MurI (retention time (RT) = 11.5) and dimeric MurI (RT = 10.6) present in the samples. The integrated areas of the peaks are written above the peaks on the SEC-HPLC spectrum.

The relative molecular mass of purified recombinant His_6_-tagged MurI was estimated by SDS–PAGE to be 31 kDa, roughly equivalent to the calculated molecular mass of 29.2 KDa. By contrast, the non-denatured MurI determined by native PAGE indicated that MurI from UA159 was in a monomeric form and a dimeric form. Therefore, SEC-HPLC, a technique for quantitative assessment of protein multimerization, was applied to monitor dimerization in MurI samples. SEC-HPLC analysis showed that the main forms of determinant were eluted at 11.5 min, 11.1 min, and 10.6 min (Fig. 4). The molecular mass of MurI was estimated to be 28.9 kDa, 38.2 kDa, and 57.7 kDa based on a calibration curve made using standard reference proteins. The peak at the elution time of 10.6 min corresponded to a homodimeric form of MurI with twice the estimated molecular mass of the main peak, which appeared at 11.5 min and was in accord with the 31 kDa monomer size determined using SDS–PAGE, indicating that the major form of MurI found in solution was a monomer. The relative abundance of the dimeric form of MurI was lower (12%) than that of the monomer form (54%). Peaks at shorter elution times at 7.4–9.5 min represented impurities of lower MW proteins, including degradation products of MurI.

### Determination of kinetic parameters

**Figure 5:**
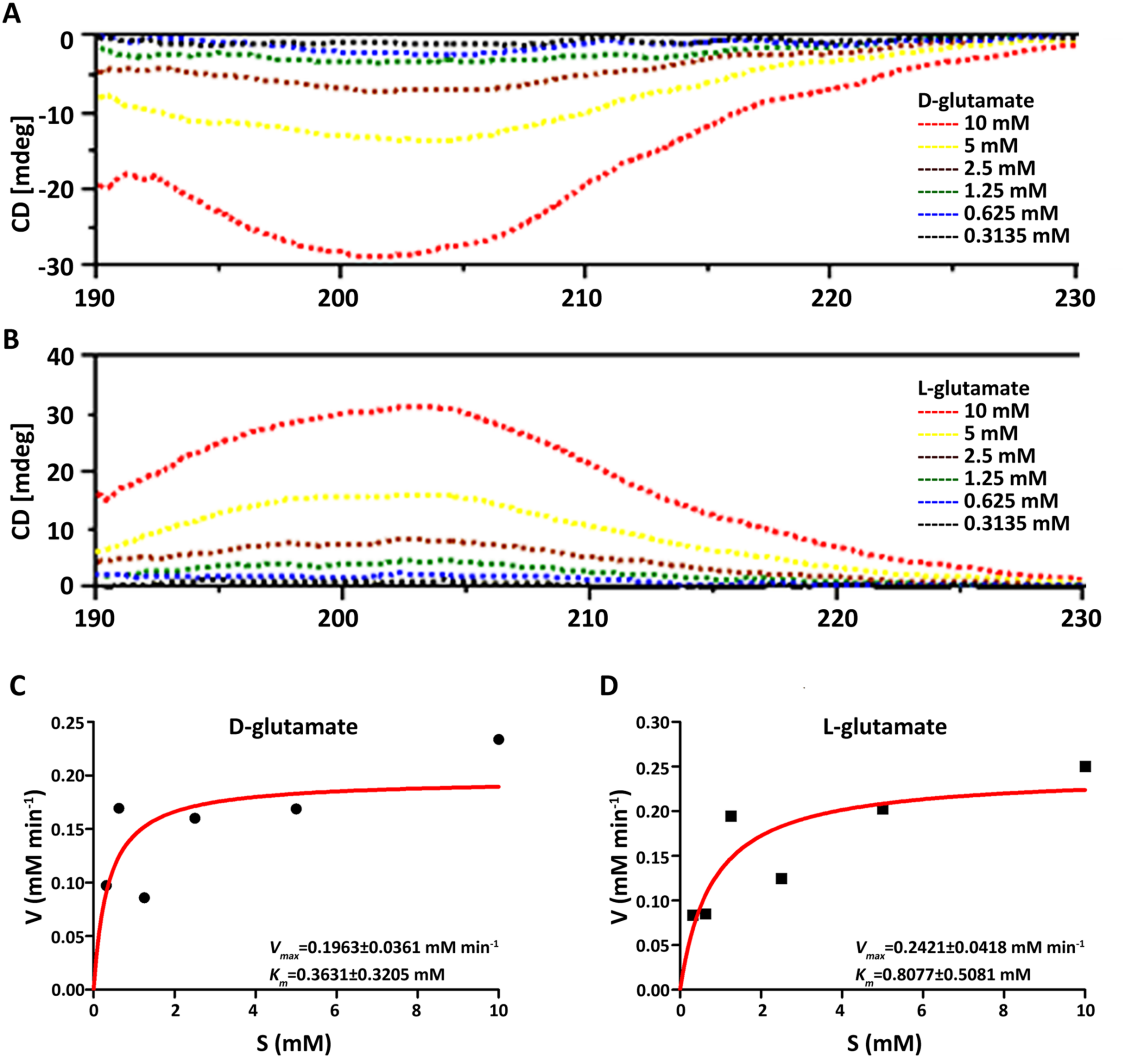
Circular dichroism (CD) spectra of *S. mutans* MurI using glutamic acids as substrates at various concentrations (A and B) and Michaelis–Menten plots for the reaction of *S. mutans* MurI examined by the CD assay (C and D). Dependence of CD on the concentrations of d- and l-glutamic acid. The CD spectra of various concentration of d-glutamic acid (A) and l-glutamic acid solution (B) were measured. Michaelis–Menten plots for calculation of *K*_m_ and *V*_max_ for MurI from *S. mutans* UA159 with d-glutamic acid (C) and l-glutamic aicd (D) as substrates.

The kinetic parameters of the purified MurI were analyzed using CD spectroscopy. The assay was performed for both of the substrates d- and l-Glutamate at various concentrations (0.3125–10 mM). Firstly, we compared the CD spectrum of d-Glu with that of l-Glu. Each CD spectrum of the d- and l-Glu solutions at various concentrations was recorded (Figs. 5A and 5B). The CD spectra of d-Glu and l-Glu displayed approximated symmetrical profiles against the *x*-axis (wavelength) in a substrate concentration-dependent manner. CD spectra of MurI in the presence of d-Glu showed one negative absorption band centered at 202 while l-Glu revealed as a positive absorption band at 204 nm. Meanwhile, the differences of curve depth change in the CD spectra between l-Glu and d-Glu might indicate that *S. mutan* MurI exhibited pseudosymmetry for the racemization of both enantiomers of glutamate in both directions. Subsequently, the kinetic constants were calculated using the observed ellipticity at 202 nm and 204 nm transformed from the maximum values of the CD spectrum. The resulting Michaelis–Menten kinetic plot is shown in Figs. 5C and 5D, and the kinetic parameters are listed in Table 2. Steady state kinetic analysis, performed for the d-Glu↔l-Glu reaction, showed a *k*_cat, l-Glu_ value of 0.0378 s^−1^ and a *k*_cat, d-Glu_ of 0.0306 s^−1^. The *K*_m, l-Glu_ is a approximately 2.22 fold higher than the *K*_m, d-Glu_, while the efficiency ratio of *k*_cat_/*K*_m(d→l)_ to *k*_cat_/*K*_m(l→d)_ was equal to 1.8 (Table 2).

**Table 2:** Kinetic parameters for purified MurI from *S. mutans* UA159 determined from Michaelis–Menten plots.

Substrate	Concentration range (mM)	Direction	*K*_m_ (mM)	*V*_max_ (mM min^−1^)	*k*_cat_ (S^−1^)	*k*_cat_/*K*_m_ (S^−1^ mM^−1^)
d-glutamate	0.3125–10	d→l	0.3631 ± 0.3205	0.1963 ± 0.0361	0.0306 ± 0.0065	0.0844 ± 0.0128
l-glutamate	0.3125–10	l→d	0.8077 ± 0.5081	0.2421 ± 0.0418	0.0378 ± 0.0056	0.0468 ± 0.0176

### The effect of temperature and pH on *S. mutans* MurI activity

**Figure 6:**
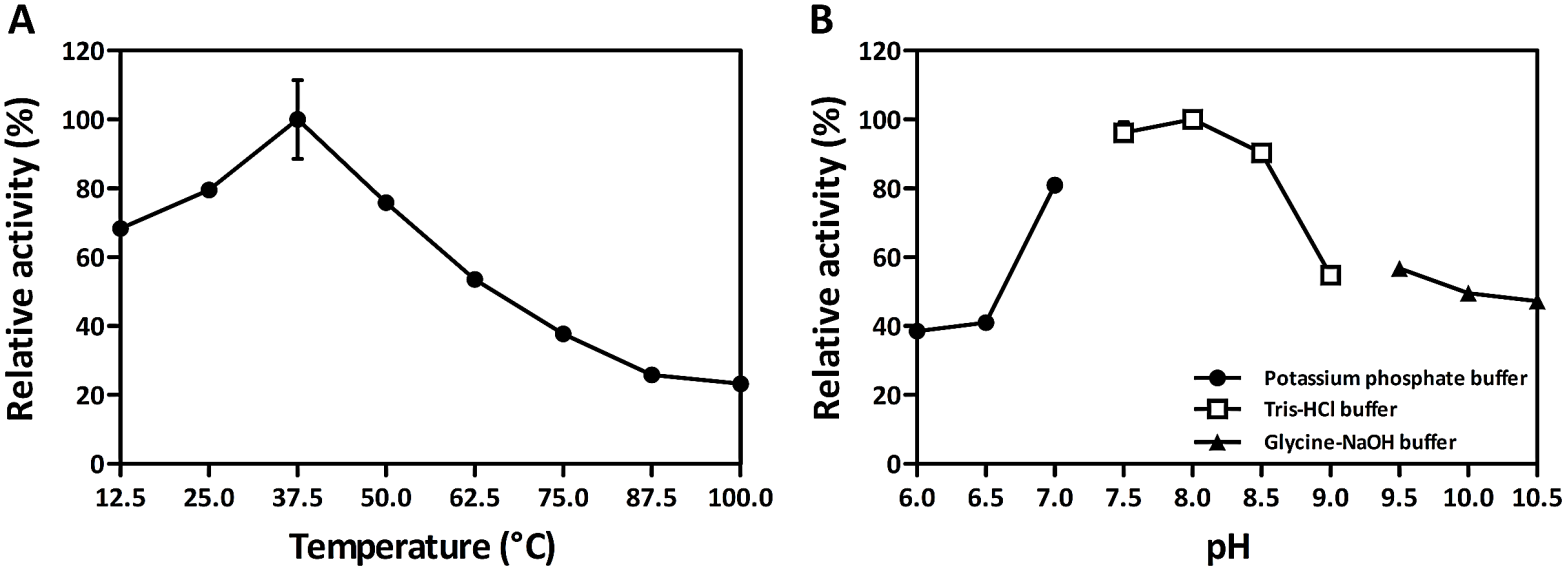
Effects of temperature (A) and pH (B) on *S. mutans* MurI activity. The optimal temperature was determined by measuring enzyme activity at various temperatures under otherwise standard assay conditions. The maximal activity, obtained at 37.5 °C, was defined as 100%. The optimal pH was measured at 30 °C in three different buffer solutions of distinct pH values under otherwise standard assay conditions. The maximal activity, obtained at pH 8, was defined as 100%. Data are shown as the means ± standard deviations (error bars; *n* = 3).

Temperature and pH were examined to determine their effect on MurI activity (Fig. 6). In order to ensure the temperature had effects solely on MurI and not on LGDH instead, the biochemical approach for measurement of relative glutamate racemase acitivIty was a couple two-reaction assay. MurI from *S. mutans* UA159 had the higher activity in a range of temperature between 25 °C and 50 °C, and exhibiting an optimal temperature of 37.5 °C with d-Glu as the substrate (Fig. 6A). Moreover, the temperature stability of MurI from *S. mutans* UA159 is shown in Fig. S5. The enzyme activity was more stable at lower temperatures than at higher temperatures. *S. mutans* MurI lost 50% of its activity when pre-incubated for 60 min at 60 °C and lost over 70% of its activity at 80 °C. The analysis to determine the optimal pH of MurI is illustrated in Fig. 6B. The pH-activity profiles for *S. mutans* MurI indicated that the enzyme exhibits a preference for high pH values, showing an optimal activity at pH comprised between 7.5 and 8.5, with its maximal activity at pH 8.0.

## Discussion

In the current study, we cloned and expressed the *murI* gene from *S. mutans* UA159 and functionally characterized the gene product in vitro, with the aim of discovering species-specific inhibitors that benefit the management of dental caries.

The comparison of the predicted structure of glutamate racemase in *S. mutans* to the discovered structure of this enzyme encoded by other bacterial species revealed quaternary structure variations, amino acid changes in the active center, and alterations in the enzyme’s substrate-binding site. Most glutamate racemases characterized in gram-positive bacteria to date are homodimeric, with the subunits associating in either head-to-head or tail-to-tail interactions (Fisher, 2008). In this study, the quaternary structure of *S. mutans* UA159 MurI, based on the computer simulation and theoretical analysis, was suggested to be a pseudo-2-fold symmetric homodimer composed of two domains of α/β protein (see Fig. 1; Fig. S1). Two conserved cysteines (Cys73 and Cys183, *S. mutans* numbering) were theoretically predicted to be located in the active-site pocket with their sulfur atoms, which are critical for catalytic activity (see Fig. 1). The equivalent residues in the *Lactobacillus fermenti* glutamate racemase are Cys73 and Cys184. Prior studies have revealed that mutation of the two cysteine residues in the *L. fermenti* enzyme to alanine eliminated activity, and their replacement with serine resulted in a 10^3^-fold reduction in activity (Glavas & Tanner, 1999). In *Bacillus anthracis*, RacE1 and RacE2 have been identified as homologous to glutamate racemase. Based on the crystal structures of d-glutamate within the dynamic sites of RacE1 and RacE2, Cys74 and Cys185 are proposed to serve as the catalytic base in abstracting the C-2 hydrogen from d-glutamate/l-glutamate in isomerization reactions (May et al., 2007). Analyses of active sites in the *H. pylori* strain revealed that the residues D7, S8, C70, and T72 in the N-terminal domain are involved in the deprotonation of d-glutamate, while the C-terminal domain encodes the residues E150, C181, T182, and H183 that participate in l-glutamate deprotonation (Glavas & Tanner, 2001). These residues, along with the conserved amino acids in the active site, have been suggested by site-directed mutagenesis to be essential for activity (Glavas & Tanner, 2001).

The high-level expression of many recombinant proteins in *E. coli* leads to the formation of aggregated proteins, commonly referred to as inclusion bodies. Inclusion bodies consist of amorphous structural, insoluble, and inactive proteins. Studies have shown that glutamate racemases accumulate into insoluble inclusion bodies when heterologously produced in *E. coli* (Kohda et al., 2002). To decrease inclusion body formation and increase the production of soluble MurI. A pilot expression experiment was conducted in a small scale (4 mL) culture with recombinant *E. coli* BL21 (DE3) harboring pCold-*murI* at different temperatures (15 °C or 37 °C). The preliminary experimental results showed overexpressed protein bands in SDS–PAGE of the cell-free extract under both low- and high-temperature conditions with acceptable solubility (Fig. S4). We did not observe the formation of inclusion bodies in the recombinant *E. coli* cells cultured in LB medium (Fig. S3). In the literature, the protein yield of glutamate racemases has been described as varying from one expression system to another due to variations in expression vector, host strain, fermentation condition, and affinity chromatography methodology. In other studies, the glutamate racemase from *Burkholderia cenocepacia* J2315 was recombinantly expressed in *E. coli* BL21 (DE3) carrying pET28a_BcGR, and the typical yield was approximately 8.0 mg of purified protein from 8 to 10 g of wet cell pellet (Israyilova et al., 2016). In addition, the glutamate racemase from *Lactobacillus plantarum* NC8 was expressed in *E. coli* BL21 (DE3) with pET20b–*murI* producing 0.4 mg of purified protein in a 4-L bioreactor cultivation after l-Glu affinity and Biofox40Q chromatography (Böhmer et al., 2013).

Glutamate racemases are heterogeneous with respect to their quaternary structure and can be monomeric, dimeric, or exist in a monomeric–dimeric equilibrium (May et al., 2007). Our SEC-HPLC analysis is in good agreement with the native PAGE results (Figs. 3 and 4) that *S. mutans* MurI fractions are roughly suggestive of monomers and dimers under native conditions. This result was consistent with some glutamate racemases from gram-positive bacteria, such as *Staphylococcus aureus* and *Enterococcus faecalis*, which are known to assemble into homodimeric structures (Lundqvist et al., 2007). Interestingly, we noted that there was a peak at 11.1 min corresponding to an estimated MW of 38.2 kDa. However, the precise oligomeric state of that portion of the protein is difficult to ascertain. Studies with mass spectrometry analysis of these protein fractions are needed to investigate their innate character. In addition, studies have shown that dimer dissociation has been responsible for an increase in catalytic rate and the dissociation of a dimer was a mechanism for enzyme activation (Mehboob et al., 2009). Based on the data in the present study, *S. mutans* MurI is predominantly a monomer in solution. The catalytic activity of *S. mutans* MurI might be due to the differences in the overall collective dynamics of the dimer and monomer. However, the oligomeric structure of MurIs in vivo is currently unknown. Further in-depth studies are needed to investigate the exact mechanisms by which *S. mutans* MurI can catalyze glutamate racemization, as well as the characteristics of endogenous MurI purified from *S. mutans* cells.

Using recombinant *S. mutans* MurI in *E. coli*, we biochemically characterized its ability to convert d-Glu to l-Glu and found that the kinetic parameters failed to fall within the same range of those of other characterized bacterial MurIs. Among the MurIs whose functions have been clarified, such as those in *Fusobacterium nucleatum* (Potrykus, Flemming & Bearne, 2009), a gram-negative anaerobe in periodontal disease, the kinetic parameters for d-glutamate have been reported with a *K*_m_ of 1.7 mM, a *k*_cat_ of 26 s^−1^, and a *k*_cat_/*K*_m_ of 15 s^−1^ mM^−1^. In *B. cenocepacia* (Israyilova et al., 2016), a gram-negative pathogen responsible for cystic fibrosis in humans, steady-state kinetic analysis showed a *K*_m_ value of 13.89 mM and a *k*_cat_ value of 1.5 min^−1^ (*scilicet* 0.025 s^−1^). In a hyperthermophilic bacterium, *Aquifex pyrophilus* (Kim et al., 1999), the *K*_m_ and *k*_cat_ values of the overexpressed glutamate racemase for the racemization of d-glutamate to the l-form were 0.50 mM and 0.25 s^−1^, respectively. The differences between our data and these three studies may have arisen due to species differences and glutamate racemase diversities. In addition, differences in methods as well as dosing protocols applied to measure kinetic parameters may also give rise to discrepancies among the investigations. Based on the multiple sequence alignments (Fig. S2) and the constructed phylogenetic tree (Fig. 3), MurIs from other strains have not been well characterized, although their genes have been sequenced and the enzymes verified as MurIs. Therefore, their enzymatic properties, such as optimal pH, temperature, and steady-state kinetic parameters cannot be compared with those of *S. mutans* MurI.

The observations that the *K*_m_ for the l-glutamate was over twofold higher than that for the d-glutamate substrate; the ratio of the kinetic parameter, *k*_cat_/*K*_m_, for the forward and reverse reaction was 0.55 for *S. mutans* MurI (see Table 2), indicate that d-glutamate binds to MurI with higher affinity and is stabilized more firmly than l-glutamate. The tendencies of the kinetic parameters noted for glutamate racemase is not specific to *S. mutans*, but come out to be a more general feature of glutamate racemases identified from other microbes in which the *K*_m_ and *k*_cat_ have been reported (May et al., 2007). It is speculated that the trends in the kinetic parameters for glutamate racemase from most organisms kinetically favor the formation of d-glutamate from l-glutamate within the cell. This is to be envisaged because, during cell wall biosynthesis, any d-glutamate formed would likely be integrated into the peptidoglycan precursors in a cost-effective manner.

## Conclusions

As shown in the current study, we isolated the gene encoding glutamate racemase from *S. mutans* UA159 and obtained heterologous expression in *E. coli*. By describing the quaternary structure, characteristics of the amino acid sequence, and enzymatic function of *S. mutans* MuI, this study not only enhances the understanding of the enzymatic properties of MurI but also may contribute to the development of structure-based drugs against pathogens causing dental caries.

## Supplemental Information

10.7717/peerj.8300/supp-1 Supplemental Information 1 Glutamate racemase (MurI) in *Streptococcusmutans*UA159. Construction of a 3D model of MurI using the *Streptococcus pyogenes* serotype M1 (SMTL ID: 2ohg.1) enzyme as a template. The template and the target have 78.41% identical residues, with a QMEAN-value of −1.01.

10.7717/peerj.8300/supp-2 Supplemental Information 2 Multiple sequence alignment and domain architecture of MurI among *S. mutans* UA159 and other known cariogenic oral streptococci. The catalytic cysteine residues are highlighted in purple. The consensus sequence in red shows the amino acids that are conserved in three or more sequences. Sequence identities (percentages) between *S. mutans* UA159 MurI and the individual enzymes are as follows: *S. mutans* GS-5, 99%; *S. salivarius*, 89.39%; *S. sanguinis*, 92.04%; *S. mitis*, 89.01%; and *Peptostreptococcus*, 65.91%.

10.7717/peerj.8300/supp-3 Supplemental Information 3 Transmission electron micrographs (TEM) analysis of inclusion bodies formation in *E. coli* BL21 upon IPTG induction. Inclusion bodies were not observed in the cytoplasm of *E. coli* cell. Images were obtained at a magnification of ×59,000.

10.7717/peerj.8300/supp-4 Supplemental Information 4 SDS–PAGE analysis of the expression and solubility of MurI in *E. coli* BL21 with or without IPTG induction. Detailed legend: Lane M1: Protein marker. Lane PC1: BSA (1 μg). Lane PC2: BSA (2 μg). Lane NC: Cell lysate without induction. Lane 1: Cell lysate with induction for 16 h at 15 °C. Lane 2: Cell lysate with induction for 4 h at 37 °C. Lane NC1: Supernatant of cell lysate without induction. Lane NC2: Debris of cell lysate without induction. Lane 3: Supernatant of cell lysate with induction for 16 h at 15 °C. Lane 4: Debris of cell lysate with induction for 16 h at 15 °C. Lane 5: Supernatant of cell lysate with induction for 4 h at 37 °C. Lane 6: Debris of cell lysate with induction for 4 h at 37 °C. The arrow indicated recombinant protein *S. mutans* MurI.

10.7717/peerj.8300/supp-5 Supplemental Information 5 Effects of temperature on *S. mutans* MurI stability. The enzyme was pre-incubated at 0, 20, 40, 60, and 80 °C for 60 min, and all measurements were performed as in Materials and Methods.

10.7717/peerj.8300/supp-6 Supplemental Information 6 Raw data exported from SWISS-MODEL applied for data analysis and preparation for Fig. 1 and Fig. S1.

10.7717/peerj.8300/supp-7 Supplemental Information 7 Raw data exported from NCBI applied for data analysis and preparation for Fig. 2 and Fig. S2.

10.7717/peerj.8300/supp-8 Supplemental Information 8 Raw data exported from SDS–PAGE image applied for data analysis and preparation for Fig. 3A.

10.7717/peerj.8300/supp-9 Supplemental Information 9 Raw data exported from Western blot image applied for data analysis and preparation for Fig. 3B.

10.7717/peerj.8300/supp-10 Supplemental Information 10 Raw data exported from Western blot image applied for data analysis and preparation for Fig. 3C.

10.7717/peerj.8300/supp-11 Supplemental Information 11 Raw data exported from native-PAGE image applied for data analysis and preparation for Fig. 3D.

10.7717/peerj.8300/supp-12 Supplemental Information 12 Raw data exported from SEC-HPLC applied for data analysis and preparation for Fig. 4.

10.7717/peerj.8300/supp-13 Supplemental Information 13 Raw CD spectra values data for kinetics analysis and preparation for Fig. 5 and Table 2.

10.7717/peerj.8300/supp-14 Supplemental Information 14 Raw absorbance values for pH effect analysis and preparation for Fig. 6.

10.7717/peerj.8300/supp-15 Supplemental Information 15 Raw absorbance values for temperature effect analysis and preparation for Fig. 6.

10.7717/peerj.8300/supp-16 Supplemental Information 16 Raw data exported from SDS–PAGE image applied for data analysis and preparation for Fig. S4.

10.7717/peerj.8300/supp-17 Supplemental Information 17 Raw absorbance values for temperature stability analysis and preparation for Fig. S5.

10.7717/peerj.8300/supp-18 Supplemental Information 18 Raw data applied for bioinformatics analysis of primary structure prediction.

## Additional Information and Declarations

### Data Availability

The following information was supplied regarding data availability: The raw data are available in the Supplemental Files.
