# Supplementary figures and images for: Heterologous expression, purification and biochemical characterization of a glutamate racemase (MurI) from Streptococcus mutans UA159
Source: PeerJ. 2019 Dec 20;7:e8300. doi: 10.7717/peerj.8300 (PMC6927343; doi:10.7717/peerj.8300)

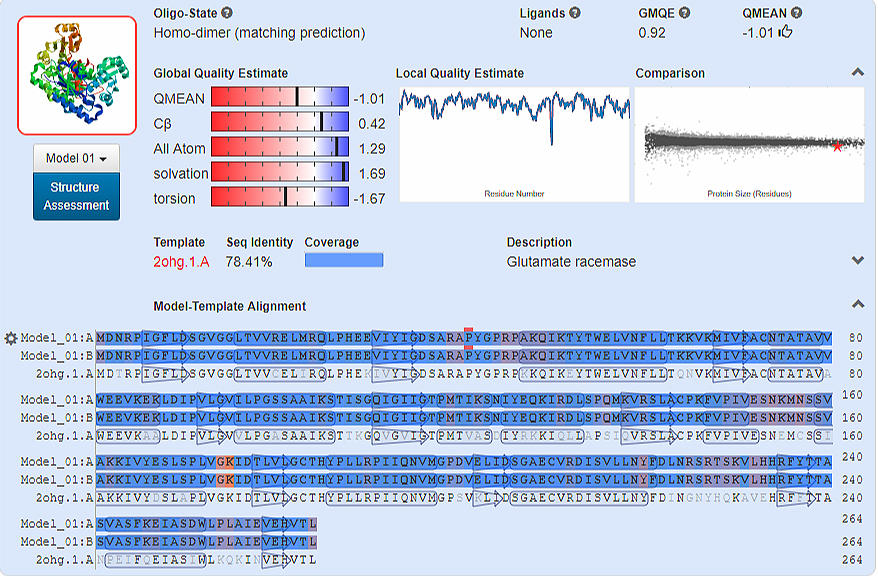

Supplement: Supplemental Information 1 — Construction of a 3D model of MurI using the Streptococcus pyogenes serotype M1 (SMTL ID: 2ohg.1) enzyme as a template. The template and the target have 78.41% identical residues, with a QMEAN-value of −1.01. [file peerj-07-8300-s001.jpg]

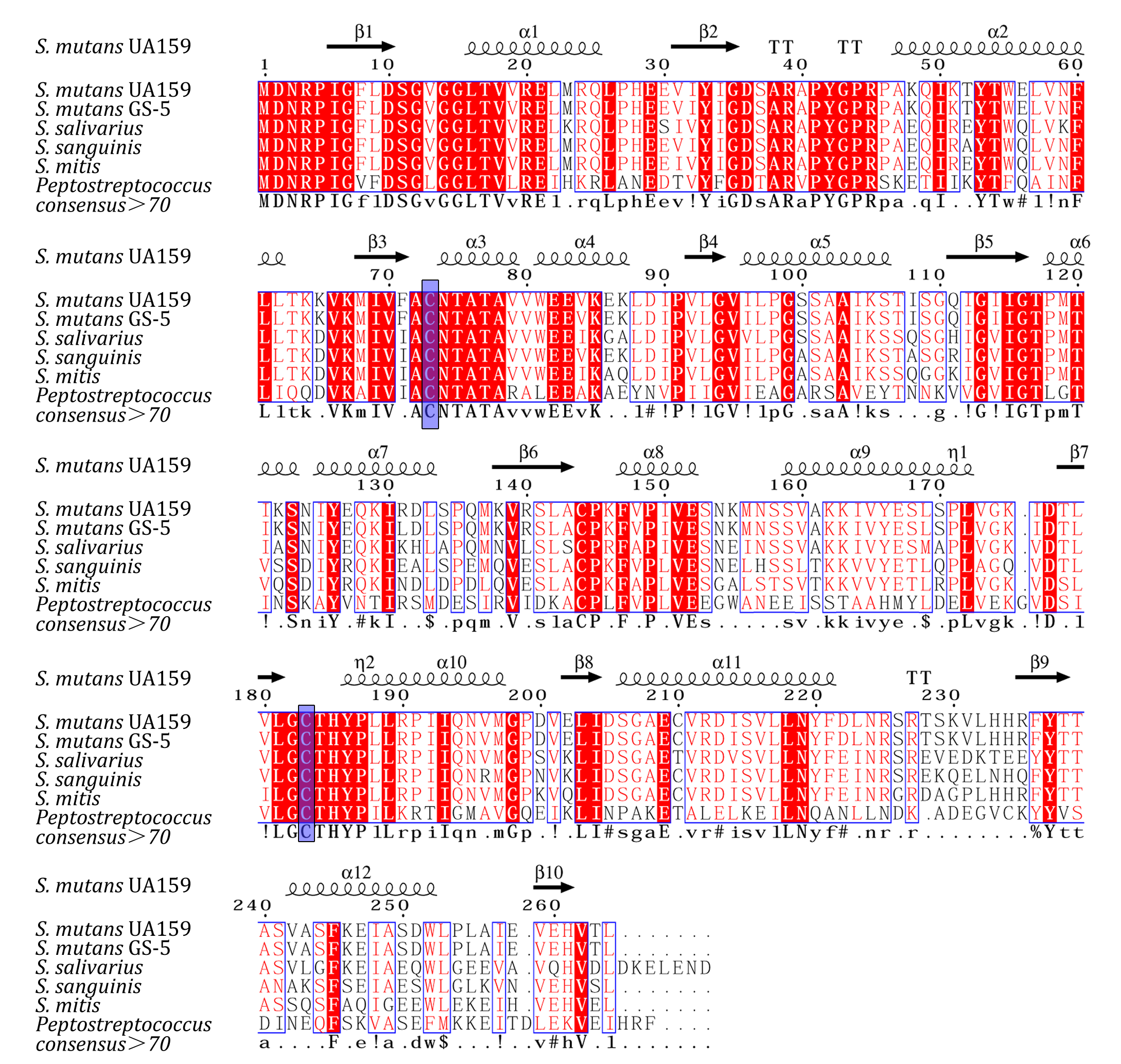

Supplement: Supplemental Information 2 — The catalytic cysteine residues are highlighted in purple. The consensus sequence in red shows the amino acids that are conserved in three or more sequences. Sequence identities (percentages) between S. mutans UA159 MurI and the individual enzymes are as follows: S. mutans GS-5, 99%; S. salivarius, 89.39%; S. sanguinis, 92.04%; S. mitis, 89.01%; and Peptostreptococcus, 65.91%. [file peerj-07-8300-s002.png]

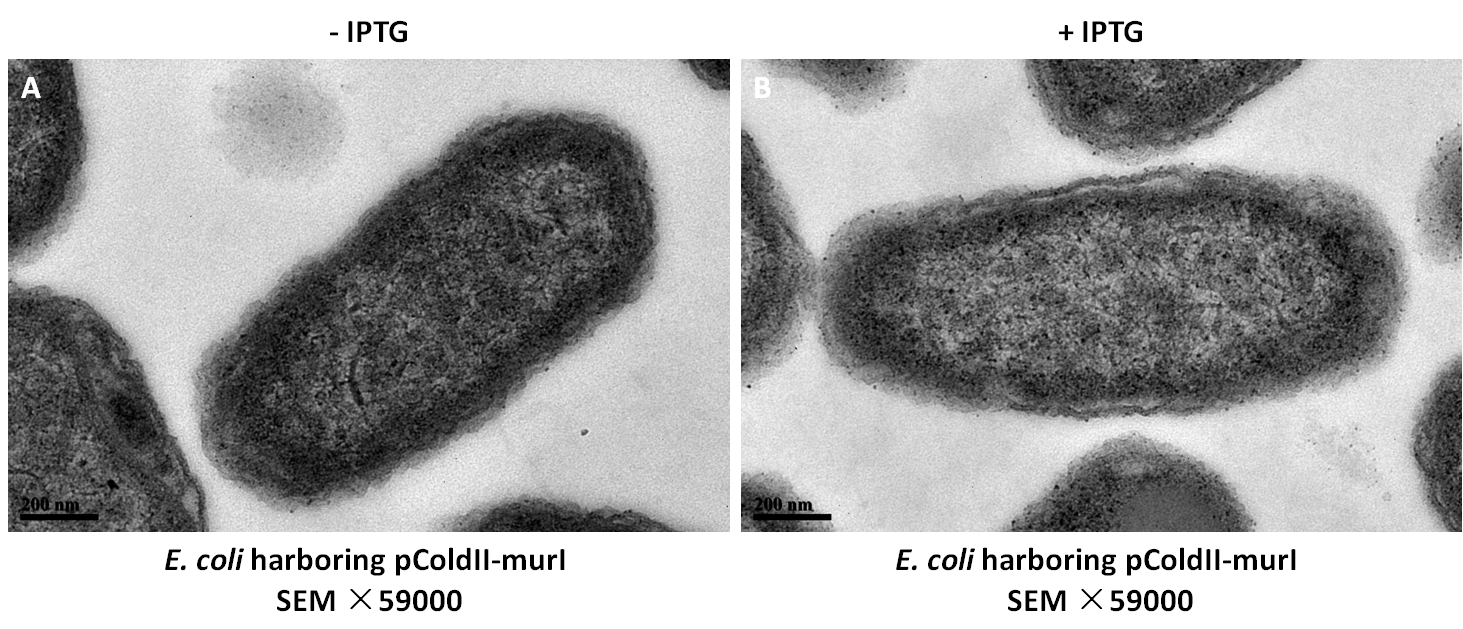

Supplement: Supplemental Information 3 — Inclusion bodies were not observed in the cytoplasm of E. coli cell. Images were obtained at a magnification of ×59,000. [file peerj-07-8300-s003.png]

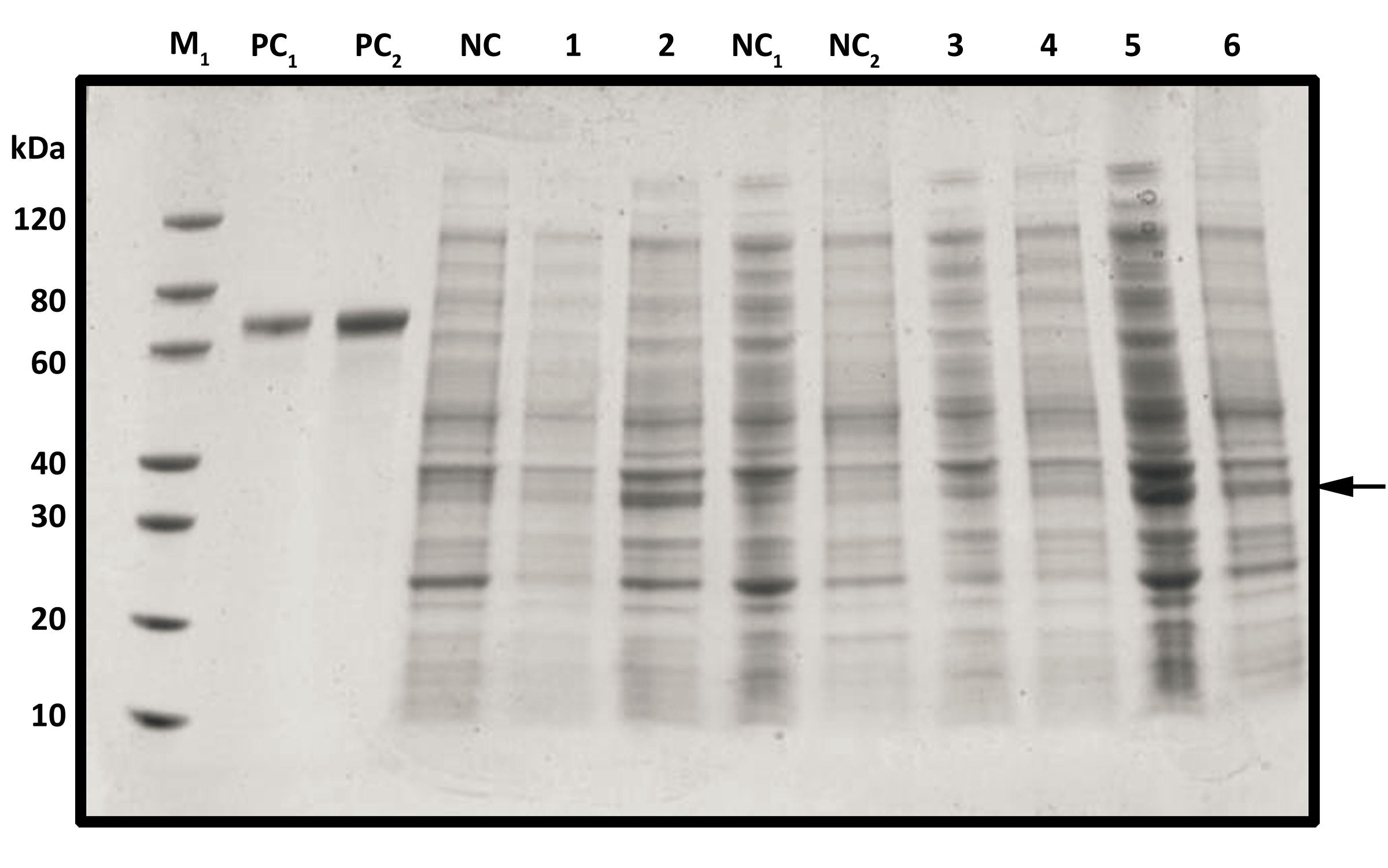

Supplement: Supplemental Information 4 — Detailed legend: Lane M1: Protein marker. Lane PC1: BSA (1 μg). Lane PC2: BSA (2 μg). Lane NC: Cell lysate without induction. Lane 1: Cell lysate with induction for 16 h at 15 °C. Lane 2: Cell lysate with induction for 4 h at 37 °C. Lane NC1: Supernatant of cell lysate without induction. Lane NC2: Debris of cell lysate without induction. Lane 3: Supernatant of cell lysate with induction for 16 h at 15 °C. Lane 4: Debris of cell lysate with induction for 16 h at 15 °C. Lane 5: Supernatant of cell lysate with induction for 4 h at 37 °C. Lane 6: Debris of cell lysate with induction for 4 h at 37 °C. The arrow indicated recombinant protein S. mutans MurI. [file peerj-07-8300-s004.png]

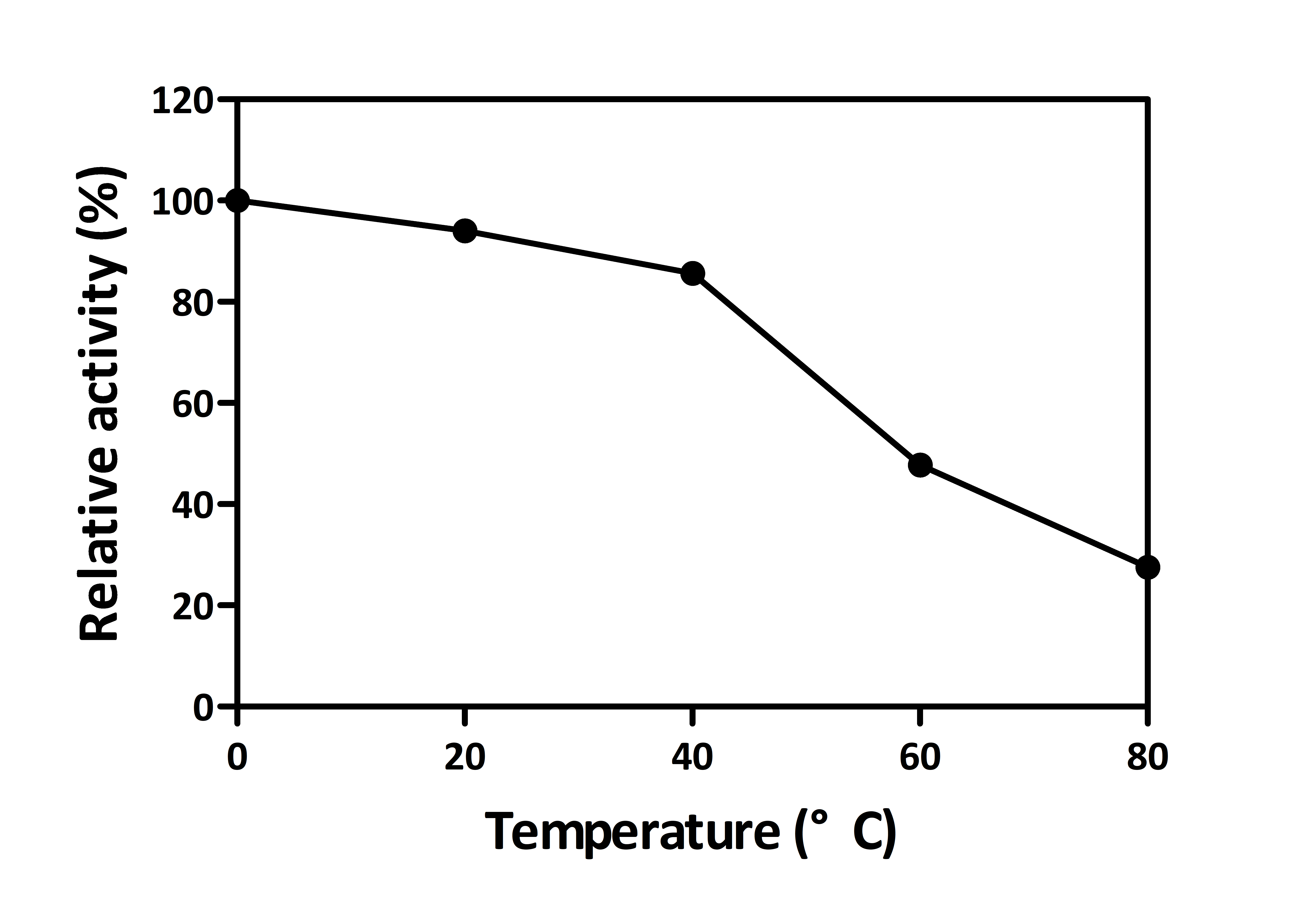

Supplement: Supplemental Information 5 — The enzyme was pre-incubated at 0, 20, 40, 60, and 80 °C for 60 min, and all measurements were performed as in Materials and Methods. [file peerj-07-8300-s005.jpg]

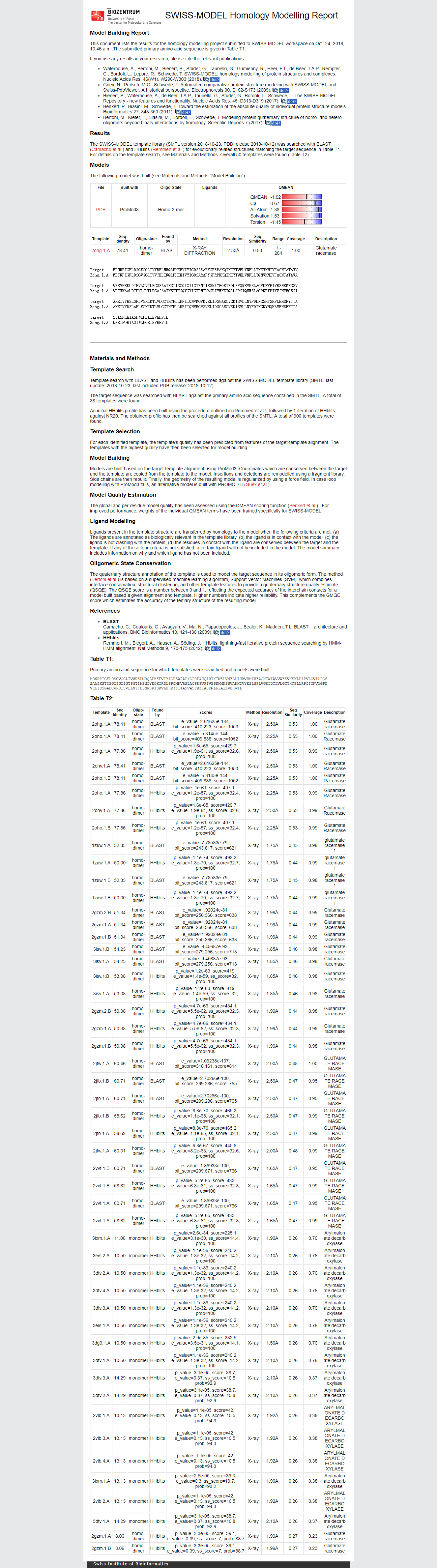

Supplement: Supplemental Information 6 [file peerj-07-8300-s006.png]

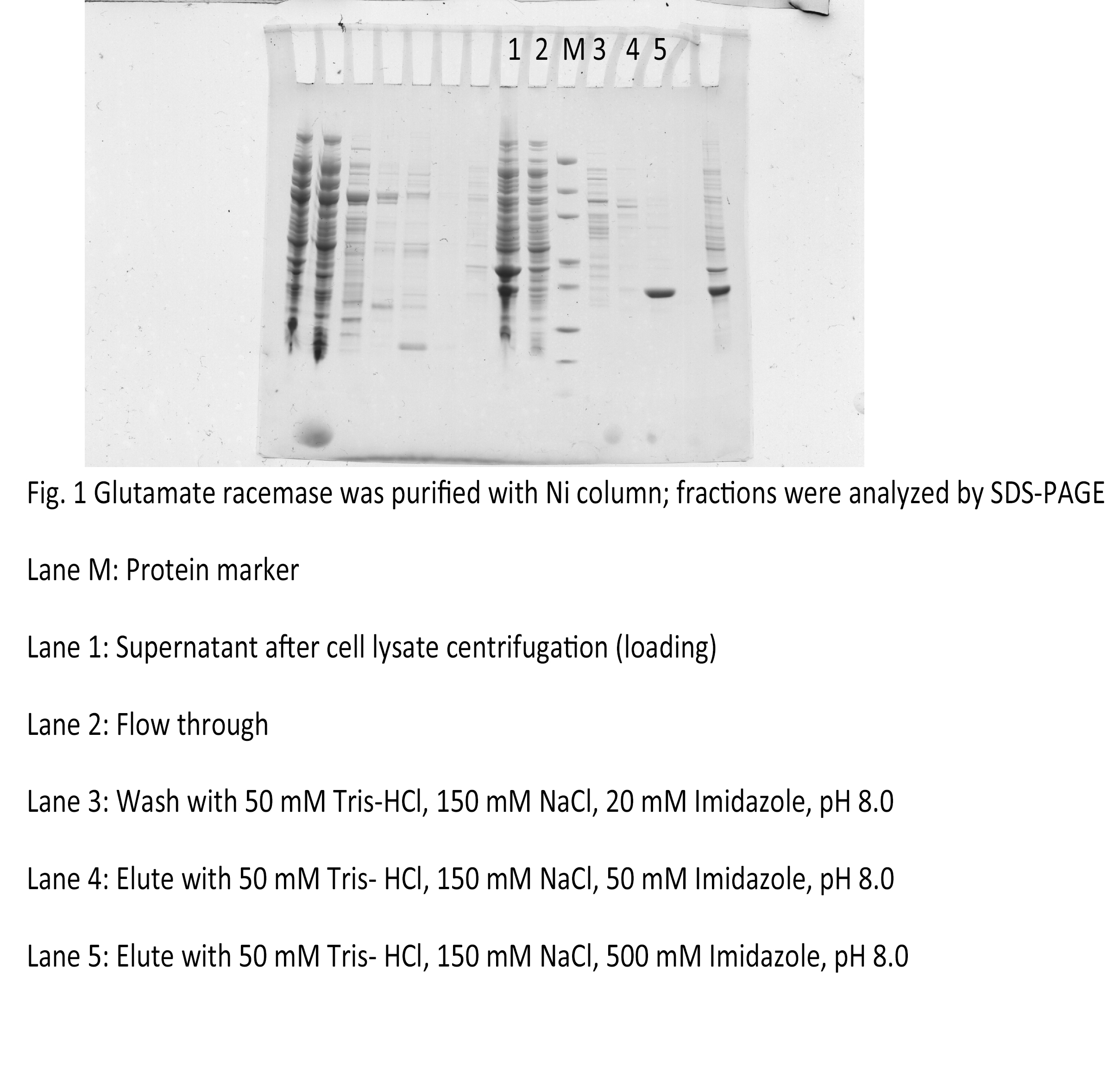

Supplement: Supplemental Information 8 [file peerj-07-8300-s008.jpg]

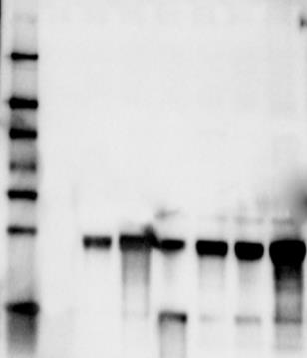

Supplement: Supplemental Information 9 [file peerj-07-8300-s009.tiff]

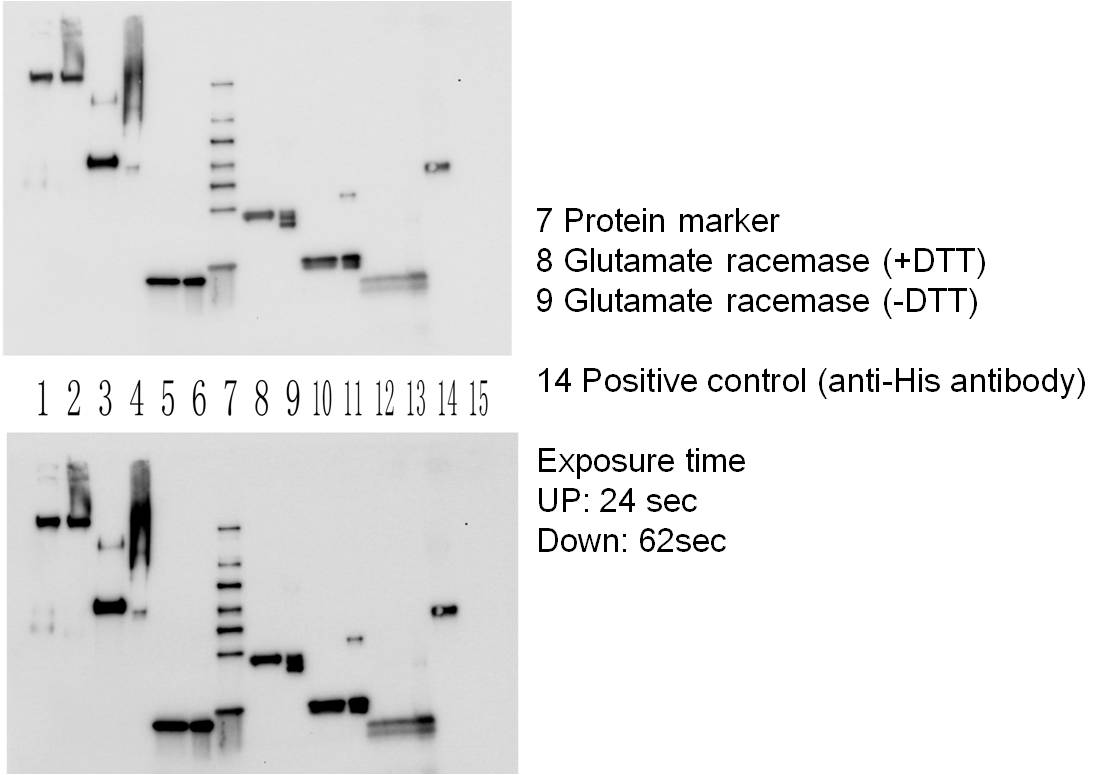

Supplement: Supplemental Information 10 [file peerj-07-8300-s010.jpg]

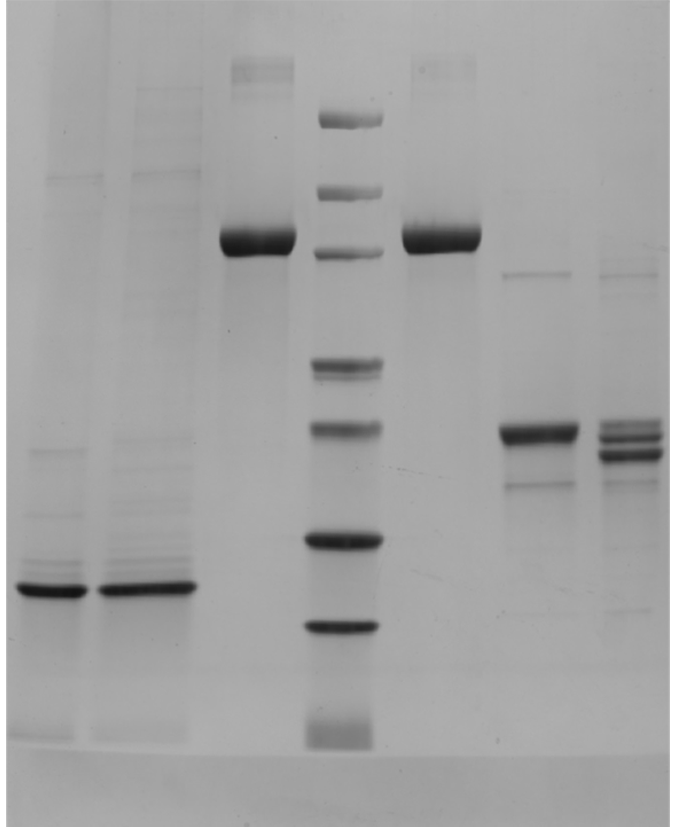

Supplement: Supplemental Information 11 [file peerj-07-8300-s011.tiff]

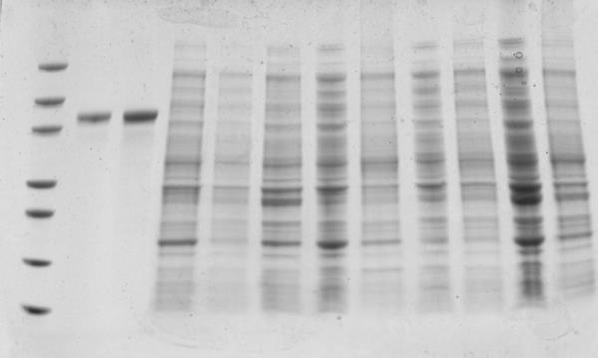

Supplement: Supplemental Information 16 [file peerj-07-8300-s016.tiff]
