# Supplementary material for: Heterologous expression, purification and biochemical characterization of a glutamate racemase (MurI) from Streptococcus mutans UA159
Source: PeerJ. 2019 Dec 20;7:e8300. doi: 10.7717/peerj.8300 (PMC6927343; doi:10.7717/peerj.8300)
Supplement: Supplemental Information 12 [file peerj-07-8300-s012.pdf]

SEC-HPLC Analysis Report

Sample Name: C6707DH130-1

Instrument: GR17010610 | Operator: fengziying

Method

Method Name: Agilent AdvanceBio SEC_0.3ml-min_25min_03082019.amx

Inj. Volume: 53.0 | Location: P1-A1

Data File: 2019-03-08 12-41-39+08-00.dx | Injection Date: 2019-03-08 12:42:36+08:00

Column Name: Agilent AdvanceBio SEC 300A | Column Size: 4.6 mm × 300 mm

Flow Rate: 0.3 ml/min | Particle Size: 2.7 μm

Mobile Phase: 0.1 mol/L Na2SO4 in 0.1 mol/L Phosphate Buffer(pH 6.7)

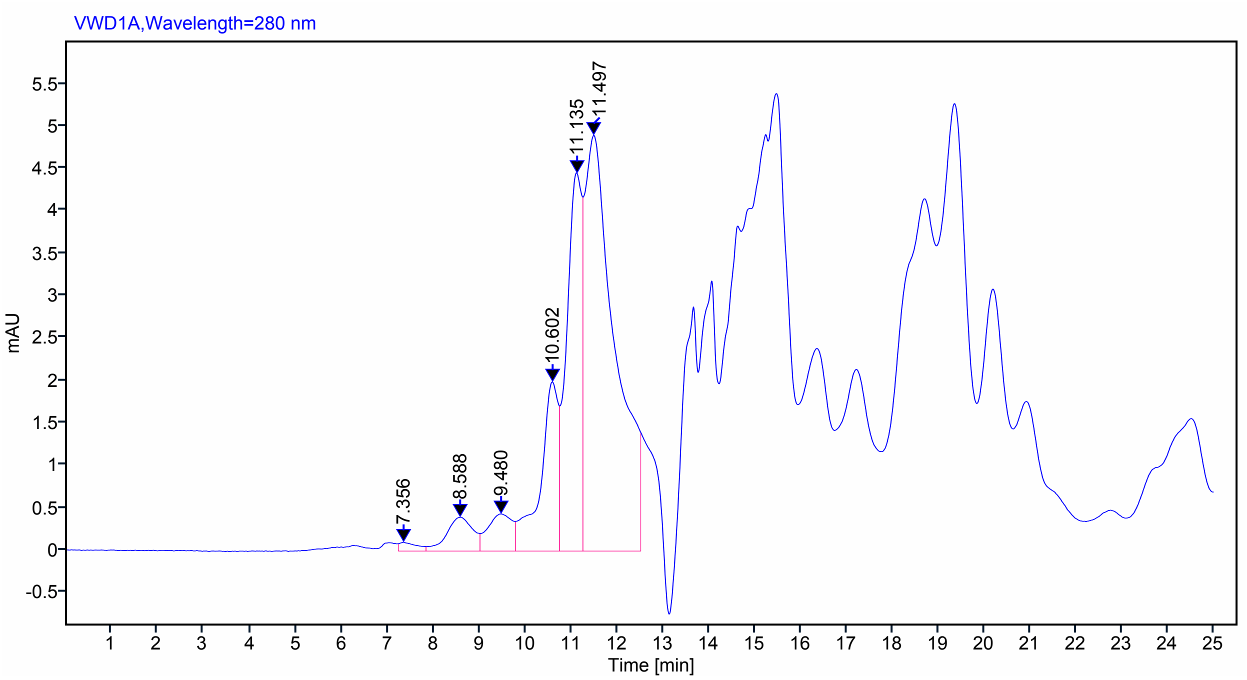

Signal: VWD1A,Wavelength=280 nm

RT [min]	Height	Width [min]	Area	Area%
7.356	0.10	0.60	2.80	0.67
8.588	0.40	1.18	15.93	3.82
9.480	0.44	0.77	16.17	3.88
10.602	1.99	0.96	53.97	12.95
11.135	4.45	0.51	102.75	24.65
11.497	4.90	1.26	225.21	54.03
		Sum	416.84	
