# Supplementary material for: Heterologous expression, purification and biochemical characterization of a glutamate racemase (MurI) from Streptococcus mutans UA159
Source: PeerJ. 2019 Dec 20;7:e8300. doi: 10.7717/peerj.8300 (PMC6927343; doi:10.7717/peerj.8300)
Supplement: Supplemental Information 18 [file peerj-07-8300-s018.docx]

# Query: AAN59353.1_putative_glutamate_racemase_[Streptococcus_mutans_UA159] (length: 264 aa)

## Sequence (MW: 29264.22 Da)

00001 MDNRPIGFLD SGVGGLTVVR ELMRQLPHEE VIYIGDSARA PYGPRPAKQI KTYTWELVNF LLTKKVKMIV

00071 FACNTATAVV WEEVKEKLDI PVLGVILPGS SAAIKSTISG QIGIIGTPMT IKSNIYEQKI RDLSPQMKVR

00141 SLACPKFVPI VESNKMNSSV AKKIVYESLS PLVGKIDTLV LGCTHYPLLR PIIQNVMGPD VELIDSGAEC

00211 VRDISVLLNY FDLNRSRTSK VLHHRFYTTA SVASFKEIAS DWLPLAIEVE HVTL

## Database searching

### NCBI Conserved Domain Database

| 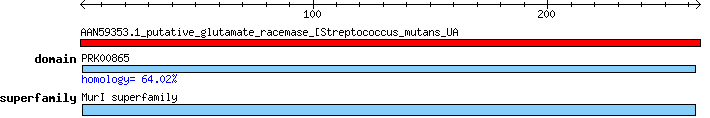 |
| --- |

### UniProt Protein Database

| 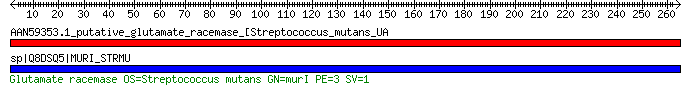 | | | | | |
| --- | --- | --- | --- | --- | --- |
| **ID** | **Description** | **Score** | **E** | **Coverage** | **Identity** |
| [Q8DSQ5](http://www.uniprot.org/uniprot/Q8DSQ5) | Glutamate racemase OS=Streptococcus mutans GN=murI PE=3 SV=1 | 1394 | 1e-153 | 100.0 % | 100.0 % |

## Isoelectric point (PI: 8.85)

| 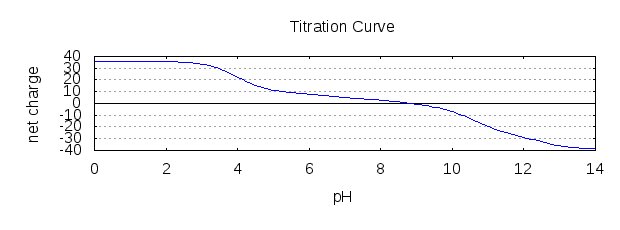 |
| --- |

## Hydropathy

| 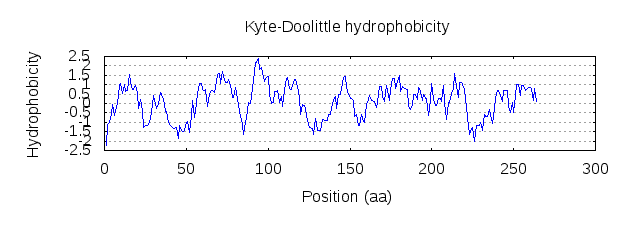 |
| --- |

## Protease cleavage

| **name** | **site** | **count** | **position** |
| --- | --- | --- | --- |
| Enterokinase | [DE][DE][DE]K[A-Z] | 0 |  |
| HRV 3C protease | LEVLFQGP | 0 |  |
| TEV protease | E[A-Z][LF]Y[FY]Q[GS] | 0 |  |
| Thrombin | [AFGILTV][AFGILTV]PR[^DE][^DE] | 0 |  |
| Thrombin | GRG | 0 |  |

## Recombinant protein solubility in E.*coli*

This protein has an **80.6** percent chance of be as **insoluble** protein when overexpressed in E. *coli*, according to the prediction by Wilkinson-Harrison statistical model.

## Protein sorting prediction

The subcellular location of this protein is predicted. It suggests that **the targeting subcellular location is not clear yet.**

## Signal peptide prediction

The signal-peptide cleavage site of this protein is predicted. It suggests that **no potential signal-peptide cleavage site is found.**

## Transmembrane region prediction

The transmembrane topology of this protein is predicted. It suggests that **the protein is not transmembrane protein.**

## Color Feature

1234567890 1234567890 1234567890 1234567890 1234567890 1234567890 1234567890

1 MDNRPIGFLD SGVGGLTVVR ELMRQLPHEE VIYIGDSARA PYGPRPAKQI KTYTWELVNF LLTKKVKMIV

signalpep .......... .......... .......... .......... .......... .......... ..........

transmem .......... .......... .......... .......... .......... .......... ..........

1234567890 1234567890 1234567890 1234567890 1234567890 1234567890 1234567890

71 FACNTATAVV WEEVKEKLDI PVLGVILPGS SAAIKSTISG QIGIIGTPMT IKSNIYEQKI RDLSPQMKVR

signalpep .......... .......... .......... .......... .......... .......... ..........

transmem .......... .......... .......... .......... .......... .......... ..........

1234567890 1234567890 1234567890 1234567890 1234567890 1234567890 1234567890

141 SLACPKFVPI VESNKMNSSV AKKIVYESLS PLVGKIDTLV LGCTHYPLLR PIIQNVMGPD VELIDSGAEC

signalpep .......... .......... .......... .......... .......... .......... ..........

transmem .......... .......... .......... .......... .......... .......... ..........

1234567890 1234567890 1234567890 1234567890 1234567890 1234

211 VRDISVLLNY FDLNRSRTSK VLHHRFYTTA SVASFKEIAS DWLPLAIEVE HVTL

signalpep .......... .......... .......... .......... .......... ....

transmem .......... .......... .......... .......... .......... ....

- Predictions are represented in the tracks below the sequence.
- symbol 's' denotes the residues located in predicted **signal** peptide.
- symbol 'm' denotes the residues located in predicted **transmembrane** segments.
